# Loss of *RAS* Mutations in Liquid Biopsies of Patients With Multi-Treated Metastatic Colorectal Cancer

**DOI:** 10.1093/oncolo/oyad299

**Published:** 2023-12-10

**Authors:** Joana Albuquerque, Diana Neto da Silva, Teresa Padrão, Luísa Leal-Costa, Rita Bizarro, Jorge Correia, Carlota Baptista, Madalena Machete, Gil Prazeres, Inês Margarido, Gonçalo Fernandes, Pedro Simões, Teresa Timóteo, Fábio Lopes, João Godinho, João Moreira-Pinto, Tânia Rodrigues, Ana Faria, Catarina Pulido, Luís Cirnes, José A Teixeira, José L Passos Coelho

**Affiliations:** Department of Medical Oncology, Hospital da Luz, Lisboa, Portugal; Department of Medical Oncology, Hospital Beatriz Ângelo, Loures, Portugal; Department of Medical Oncology, Hospital da Luz, Lisboa, Portugal; Department of Medical Oncology, Hospital Beatriz Ângelo, Loures, Portugal; Department of Medical Oncology, Hospital Beatriz Ângelo, Loures, Portugal; Department of Medical Oncology, Hospital da Luz, Lisboa, Portugal; Department of Medical Oncology, Hospital Beatriz Ângelo, Loures, Portugal; Department of Medical Oncology, Hospital Beatriz Ângelo, Loures, Portugal; Department of Medical Oncology, Hospital da Luz, Lisboa, Portugal; Department of Medical Oncology, Hospital da Luz, Lisboa, Portugal; Department of Medical Oncology, Hospital da Luz, Lisboa, Portugal; Department of Medical Oncology, Hospital Beatriz Ângelo, Loures, Portugal; Department of Medical Oncology, Hospital da Luz, Lisboa, Portugal; Department of Medical Oncology, Hospital da Luz, Lisboa, Portugal; Department of Medical Oncology, Hospital Beatriz Ângelo, Loures, Portugal; Department of Medical Oncology, Hospital da Luz, Lisboa, Portugal; Department of Medical Oncology, Hospital Beatriz Ângelo, Loures, Portugal; Department of Medical Oncology, Hospital da Luz, Lisboa, Portugal; Department of Medical Oncology, Hospital Beatriz Ângelo, Loures, Portugal; Department of Medical Oncology, Hospital da Luz, Lisboa, Portugal; Department of Medical Oncology, Hospital da Luz, Lisboa, Portugal; Department of Medical Oncology, Hospital Beatriz Ângelo, Loures, Portugal; Department of Medical Oncology, Hospital da Luz, Lisboa, Portugal; Instituto de Patologia e Imunologia Molecular da Universidade do Porto (IPATIMUP), Porto, Portugal; Department of Medical Oncology, Hospital Beatriz Ângelo, Loures, Portugal; Department of Medical Oncology, Hospital da Luz, Lisboa, Portugal

**Keywords:** metastatic colorectal cancer, *RAS* mutations, Neo*RAS* wild type, liquid biopsy

## Abstract

**Background:**

Liquid biopsy (LB) is a non-invasive tool to evaluate the heterogeneity of tumors. Since *RAS* mutations (*RAS*-mut) play a major role in resistance to antiepidermal growth factor receptor inhibitors (EGFR) monoclonal antibodies (Mabs), serial monitoring of *RAS*-mut with LB may be useful to guide treatment. The main aim of this study was to evaluate the prognostic value of the loss of *RAS*-mut (Neo*RAS*-wt) in LB, during the treatment of metastatic colorectal cancer (mCRC).

**Methods:**

A retrospective study was conducted on patients with mCRC between January 2018 and December 2021. *RAS*-mut were examined in tissue biopsy, at mCRC diagnosis, and with LB, during treatment.

**Results:**

Thirty-nine patients with *RAS*-mut mCRC were studied. LB was performed after a median of 3 lines (0-7) of systemic treatment including anti-vascular endothelial growth factor (anti-VEGF) Mabs. *NeoRAS*-wt was detected in 13 patients (33.3%); 9 (69.2%) of them received further treatment with anti-EGFR Mabs with a disease control rate of 44.4%. Median overall survival (OS), from the date of LB testing, was 20 months in the Neo*RAS*-wt group and 9 months in the persistent *RAS*-mut group (log-rank 2.985; *P* = .08), with a 12-month OS of 84.6% and 57.7%, respectively. *NeoRAS*-wt was identified as a predictor of survival (HR = 0.29; *P* = .007), with an 11-month improvement in median OS and a 71% decrease in risk of death, in heavily pretreated patients

**Conclusions:**

In conclusion, monitoring clonal evolution in mCRC by LB may provide an additional treatment line for patients with Neo*RAS*-wt in advanced disease.

## Teaser

This study evaluated the prognostic value of the loss of RAS mutation occurrence in liquid biopsy in the treatment of metastatic colorectal cancer.

**Implications for Practice**

Performing liquid biopsies in patients with *RAS*-mutated tumors should be considered, preferably early in the course of the disease, as loss of mutation represents an opportunity for treatment with antiepidermal growth factor receptor inhibitors Mabs (otherwise unlikely), with an apparent overall survival benefit.

## Introduction

Metastatic colorectal cancer (mCRC) accounts for 25% to 30% of all cases of CRC, with 1.2 million estimated new cases per year.^1-3^ The overall prognosis remains poor, with a 5-year overall survival (OS) lower than 20%.^4^

Treatment options for patients with mCRC are limited and depend on the mutational status of specific genes. In this context, tissue-based biomarkers such as *KRAS, NRAS, BRAF V600E* mutations, *HER2* mutations or amplification and microsatellite instability, predict the efficacy of systemic therapy and prognosis.^4-6^ Since *RAS* mutated (RAS-mut) patients with mCRC do not benefit from treatment with antiepidermal growth factor receptor inhibitors (anti-EGFR) monoclonal antibodies (MAbs),^4,7-11^ the outcomes are worse than in *RAS* wild-type (wt) mCRC (median OS of 25 vs. 37 months and progression-free survival [PFS] of 10.9 vs. 12.8 months, respectively).^6,12^

Tumor heterogeneity is hypothesized to play a critical role in the limited prognosis of mCRC. Selective mechanisms during treatment can result in significant changes in tumor biology.^13,14^ The analysis of circulating tumor DNA (ctDNA), through its ability to reproduce tumor heterogeneity, is a surrogate of tumor biopsy for detecting mutations.^5,15,16^ This technique has the advantages of being less invasive than a tissue biopsy and easily repeated over time.^16^

Studies with liquid biopsies (LB) have been mainly focused on the emergence of *RAS*-mut clones, under the selective pressure of anti-EGFR Mabs in patients with *RAS*-wt mCRC, providing a molecular rationale for LB-based adaptive therapies.^4^ On the other hand, studies with LB monitoring molecular changes during the treatment course of *RAS*-mut mCRC have reported a negative selection of *RAS-*mut clones in ctDNA, in a rate of 37% to 57%, suggesting an unpredicted clonal shift of *RAS*-mut clones during standard systemic treatment, the Neo*RAS*-wt concept. This phenomenon has not been completely explained, and it is unclear whether it is a true clonal shift or a false negative result due to the low analytic sensitivity of some LB assays and/or to the absence of *RAS*-mut circulating tumor DNA. The doubts are reasonable, since the development of *RAS* mutation is an early event in colorectal carcinogenesis. This topic has been addressed in a study of Nicolazzo C et al, in which detection of tumor-specific DNA methylation alterations in ctDNA has been suggested as a specific tool to confirm the presence of tumor DNA in plasma. The methylation test confirmed the presence of ctDNA in *RAS-*wt samples, at the time of disease progression, thus corroborating that the negative selection of *RAS*-mut clones during the clonal evolution of *RAS-mut* colorectal cancer is neither a rare event nor a false negative result. Due to the intriguing biological consequences of the Neo*RAS-*wt phenomenon, tracking the dynamic evolution of gain or loss of *RAS*-mut clones with LB, along the course of mCRC disease, may impact its therapeutic management.^6,7,12^

Subsequent treatment of these patients with anti-EGFR Mabs is increasingly being discussed and led to the design of several retrospective and prospective non-randomized studies, aiming to investigate the efficacy and safety of anti-EGFR Mabs treatment [in monotherapy or in combination with chemotherapy (ChT)], in previously *RAS*-mut patients with mCRC who converted to *NeoRAS*-wt, as assessed by ctDNA analysis.^16^ In this setting, Bouchahda et al reported a promising 50% objective response rate, including 2 patients with complete response and 4 with partial response after a median of 6 cycles of cetuximab plus FOLFIRI.

The aim of the present study was to evaluate the prognostic impact of *NeoRAS-*wt occurrence in LB and identify patient and tumor characteristics that correlate with *NeoRAS-wt.*

## Materials and Methods

### Study Design

This retrospective observational study was conducted in 2 Portuguese hospitals (Hospital da Luz and Hospital Beatriz Ângelo). Patients with mCRC with *RAS-mut*, documented in the last tumor biopsy, were eligible for monitoring by LB. Patients with disease progression, after receiving standard systemic treatment, and with Neo*RAS*-wt in LB were discussed at tumor board, and subsequent treatment was chosen based on published evidence, characteristics of patients and tumor, and toxicity of treatment, in a shared decision between patients, families, and physicians. The primary endpoint was OS in patients with mCRC with *NeoRAS*-wt (in LB collected during treatment) compared with patients with persistent *RAS-mut*. OS was defined as the time from LB collection to death or last follow-up.

Other exploratory endpoints were: (1) Disease Control Rate (DCR) with anti-EGFR Mabs in *NeoRAS*-wt group, defined as the rate of patients with complete response, partial response, or stable disease per RECIST 1.1 criteria; (2) impact in OS of: (a) Eastern Cooperative Oncology Group Performance Status (ECOG PS) (0, ≥1), (b) tumor sidedness (left vs. right colon vs. rectum), (c) TNM stage at diagnosis (I, II, III, or IV according to American Joint Committee on Cancer [AJCC] 8th edition), (d) number of metastatic sites (1 or ≥2 sites), (e) site of metastases (hepatic, pulmonary, peritoneal, or other sites), (f) level of carcinoembryonic antigen (CEA) at time of the diagnosis of metastatic disease (normal [<3.5 ng/mL] or high [≥3.5 ng/mL]), (g) time to first progression after systemic treatment for mCRC (above vs. below the median of 11 months), (h) number of treatment lines performed before LB (<3 or ≥3), and (i) *NeoRAS*-wt occurrence.

Efficacy of treatment was evaluated using imaging exams requested at the choice of physician, reviewed by the radiologist of the institutions, and classified as complete response, partial response, stable disease, or progression disease according to RECIST 1.1 criteria. No repeat confirmatory diagnostic imaging procedures were required.

Right-side colon cancers (RSCC) were considered tumors of the cecum, ascending colon, hepatic flexure, or transverse colon; left-side colon cancers (LSCC) included tumors on the splenic flexure, descending or sigmoid colon; rectal cancer was defined as tumors within ≤15 cm from the anal margin.

Regarding CEA levels, reference values of a local laboratory were used as the cutoff for “normal” or “high” level classification. For the number of metastatic sites, time to first progression to systemic treatment for mCRC, and number of treatment lines performed before LB, the cutoffs were calculated using the median values for each variable in the study population.

Time to first progression to systemic treatment for mCRC was defined as the time from starting first-line systemic treatment of metastatic disease until progression.

### Patients

Between January 2018 and December 2021, all patients with mCRC who had a LB throughout their treatment were screened. Inclusion criteria were: (1) age ≥ 18 years, (2) histologically confirmed CRC, (3) presence of *RAS-mut* in tumor, and (4) available results of *RAS* and *BRAF* mutational status by LB performed during the study period. Exclusion criteria were: (1) histology other than adenocarcinoma not-otherwise-specified (NOS), mucinous adenocarcinoma, or signet ring cell carcinoma; (2) previous treatment with anti-EGFR Mabs; (3) second cancer under investigation or treatment at the time of diagnosis; and (4) absence of follow-up data in electronic medical records (EMR).

### Genetic and Biochemical Analysis

Blood samples were collected using a blood collection tube with formaldehyde-free preservative stabilizing stabilized nucleated blood cells. Molecular evaluation of mutations in genes *KRAS* and *NRAS* genes (codons 12, 13, 59, 61, 117, and 146) and in codon 600 of the *BRAF* gene (MN_004333.4) was carried out using the Idylla method (Biocartis), with a multiplex PCR reaction. The overall reported tissue-plasma concordance of this test was 78.9%. The analytical sensitivity was ≤1% for mutations in exons 2 and 3 of *KRAS* and in codon 600 of the *BRAF* gene; and ≤5% for mutations in exon 4 of the *KRAS* and exons 2, 3, and 4 of the *NRAS* gene.^17-20^ To avoid false negative results, quality control was used in LB specimens: values greater than 26 and 35.5 for *KRAS* and *NRAS/BRAF*, respectively, suggested low amounts of ctDNA, and thus, a negative result did not exclude the presence of mutations in these genes. In these cases, a new sample (blood or tumor tissue) was obtained, and the molecular test was repeated.

Due to the retrospective nature of the study, LB was performed throughout the treatment, with no specific schedule, according to the physician’s choice of timing.

CEA was determined in peripheral blood by a chemiluminescence immunoassay.

### Data Collection

Data were collected by the authors from the hospital EMR.

Collected demographic and clinical variables were: gender, age at diagnosis, ECOG PS (0, ≥1), at diagnosis and at time of LB collection, date of diagnosis of CRC and mCRC, CRC location (right colon, left colon, and rectum), TNM stage (according to the AJCC 8th edition), number of metastatic sites (1, ≥2), and sites of metastases (lung, liver, peritoneum, others), at diagnosis and at time of LB collection, CEA levels at diagnosis and at the time of first relapse and at the time of LB collection (<3.5 ng/mL; ≥3.5 ng/mL), date of first relapse, date of relapse after treatment according to LB result, date of death and of last follow-up.

Collected treatment and pathologic variables were: date of surgery, type of surgery (primary tumor resection, primary tumor resection, and metastasectomy or other), intent of primary treatment (palliative or curative), adjuvant ChT (regimen), treatment with antiangiogenic agents and/or anti-EGFR Mabs before LB, number of treatment lines before LB (<3, ≥3), response and duration of response to anti-EGFR Mabs in *NeoRAS-*wt population, treatment regimen after LB, duration of treatment response after LB, number of total lines of treatment, histological subtype, World Health Organization (WHO) histological grade (low or high grade), *RAS* and *BRAF* mutations, and *HER2* mutation or amplification in tumor tissue, *RAS* and *BRAF* mutations in LB.

Data from patients lost to follow-up were considered up until the time of the last physical evaluation, after which, patients were censored for survival analysis.

### Ethical Considerations

This study was approved by the Institutional Review Board/Independent Ethics Committee (IRB/IEC) of both centers and designed according to Good Clinical Practice guidelines and the Declaration of Helsinki. Waiver of informed consent was requested by the investigators and accepted by the IRB/IEC owing to the retrospective and non-interventional nature of the study. Clinical data were treated with pseudonymization and kept accessible only to the primary investigators.

### Statistical Analysis

The statistical analysis included descriptive statistical measures (absolute and relative frequency, mean and standard deviation) and inferential statistics.

Comparison of variables between *NeoRAS*-wt and persistently *RAS*-mut mCRC groups in LB was performed using Mann-Whitney test for continuous variables, and Pearson’s chi-square or Fisher’s exact tests for categorical variables.

The influence of the collected variables on the *NeoRAS*-wt phenomenon was assessed with binomial logistic regression. *NeoRAS*-*wt* occurrence was the dependent variable, the independent variables included in the model were ECOG PS, primary tumor location, TNM stage, number of metastatic sites, site of metastases, CEA level, time to first progression to systemic treatment for mCRC, and number of treatment lines before LB. All variables tested were previously described as factors that could influence response to treatment and clonal selection. A top-down approach (univariate *P* < .11) was used to obtain the final model.

Standard survival analysis was performed for OS using Kaplan-Meier estimates, log-rank test, and multivariate Cox proportional-hazards regression model. All multivariate models included primary tumor location, TNM stage, number of metastatic sites and site of metastases, CEA level, time to first progression to systemic treatment for mCRC, number of treatment lines before LB, and occurrence of *NeoRAS*-wt, as variables of interest; other possible confounders were selected using a top-down approach (univariate *P* < .11).

The overall alpha level in this study was .05. Analyses were performed using SPSS Statistics for Windows, Version 28.0 (Armonk, NY, USA: IBM Corp.)

## Results

### Patients

**Figure 1.**
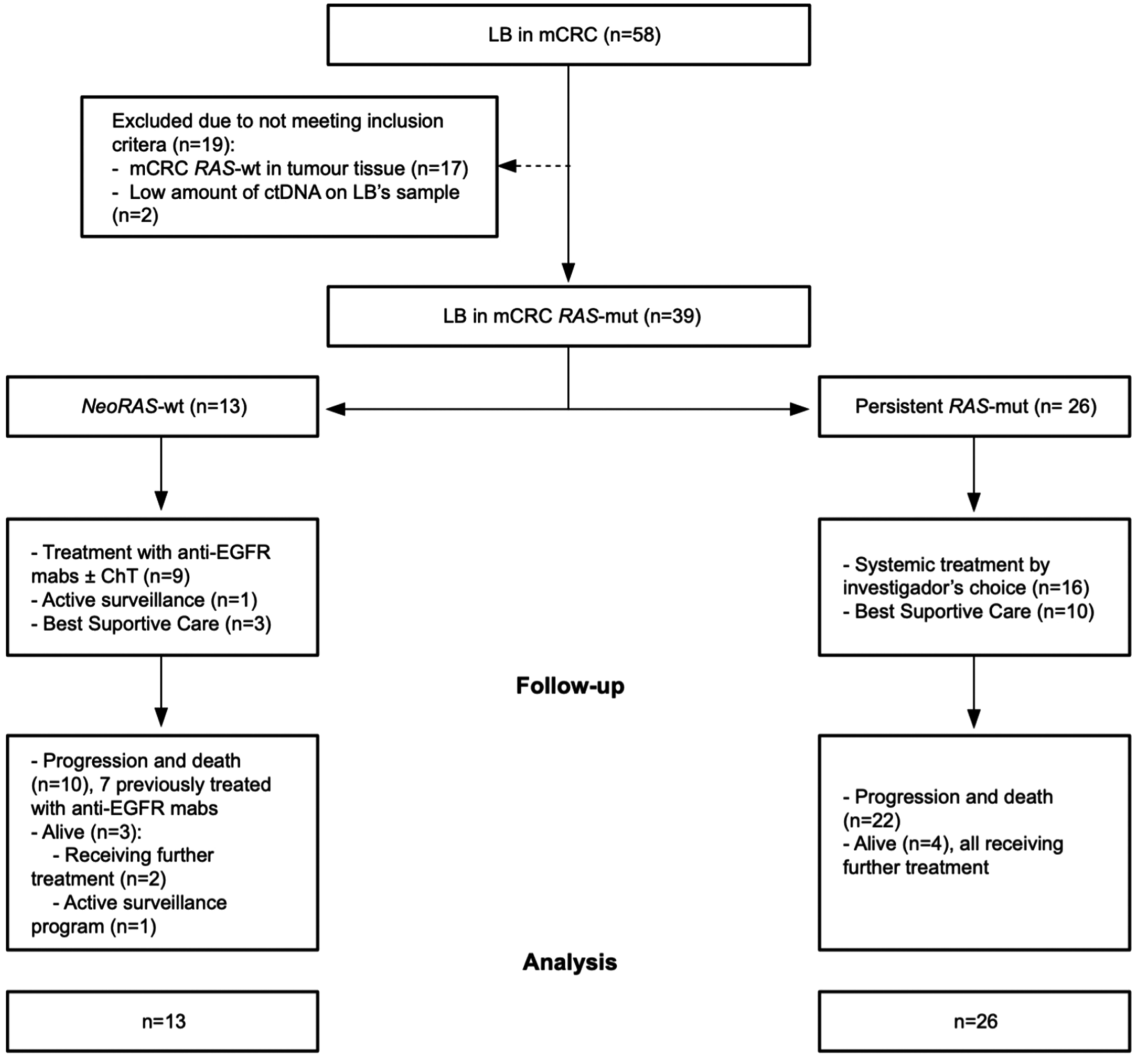
CONSORT diagram. Abbreviations: ChT: chemotherapy; ctDNA: circulating tumor DNA; anti-EGFR: epidermal growth factor receptor inhibitors; LB: liquid biopsy; mCRC: metastatic colorectal cancer; *RAS*-mut: *RAS* mutated; *RAS*-wt: *RAS* wild type.

From January 2018 to December 2021, a total of 58 LB were performed; 39 were in patients with *RAS*-mut in tumor tissue fulfilling the study eligibility criteria: 13 (33.3%) were *NeoRAS-wt*, and 26 (66.7%) were persistent *RAS*-mut (Table 1 and Fig. 1).

**Table 1.** Demographic and clinical characteristics of the population.

Variable	Overall population (n = 39)	RAS-mut (n = 26)	NeoRAS-wt (n = 13)	P-value^*^
Age (median, range)	63 (41-86)	64 (41-86)	64 (41-86)	.648
ECOG PS (N, %)				.447
0	30 (76.9%)	21 (80.8%)	9 (69.2%)	
≥1	9 (23.1%)	5 (19.2%)	4 (30.8%)	
Gender (N, %)				.482
Female	14 (35.9%)	8 (30.8%)	6 (46.2%)	
Male	25 (64.1%)	18 (69.2%)	7 (53.8%)	
Primary tumor location (N, %)				.084
Left colon	13 (33.3%)	11 (42.3%)	2 (15.4%)	
Right colon	18 (46.2%)	12 (46.2%)	6 (46.2%)	
Rectum	8 (20.5%)	3 (11.5%)	5 (38.5%)	
WHO histological grade (N, %)				1.000
Low grade	26 (66.7%)	19 (73.1%)	7 (53.8%)	
High grade	4 (10.3%)	3 (11.5%)	1 (7.7%)	
Unknown	*9 (23.1%)*	*4 (15.4%)*	*5 (38.5%)*	
Stage at diagnosis^**^ (N, %)				**.019**
II	1 (2.6%)	1 (3.8%)	0 (0%)	
III	15 (38.5%)	6 (22.1%)	9 (69.2%)	
IV	23 (59.0%)	19 (73.1%)	4 (30.8%)	
Number of metastatic sites^†^ (N, %)				.090
1 site	23 (59.0%)	18 (69.2%)	5 (38.5%)	
≥2 sites	16 (41.0%)	8 (30.8%)	8 (61.5%)	
Metastatic sites (N, %)				
Liver	25 (64.1%)	19 (73.1%)	6 (46.2%)	.157
Lung	12 (30.8%)	7 (26.9%)	5 (38.5%)	.486
Peritoneum	12 (30.8%)	7 (26.9%)	5 (38.5%)	.486
Other	9 (23.1%)	4 (15.4%)	5 (38.5%)	.256
Type of surgery (N,%)				.158
Primary tumor alone	26 (66.7%)	15 (57.7%)	11 (84.6%)	
Primary tumor + metastasectomy	12 (30.8%)	10 (38.5%)	2 (15.4%)	
Missing	*1 (2.6%)*	*1 (3.8%)*	*0 (0)*	
Level of CEA at metastatic time (N, %)				.467
<3.5ng/mL	15 (38.5%)	9 (34.6%)	6 (46.2%)	
≥3.5 ng/mL	20 (51.3%)	15 (57.7%)	5 (38.5%)	
Missing	*4 (10.3%)*	*2 (7.7%)*	*2 (15.4%)*	
Number of previous treatment lines before LB^‡^ (N,%)				.486
<3 lines	12 (30.8%)	7 (26.9%)	5 (38.5%)	
≥3 lines	27 (69.2%)	19 (73.1%)	8 (61.5%)	
Time to first progression of systemic treatment in mCRC^§^				.320
<11 months	19 (48.7%)	11 (42.3%)	8 (61.5%)	
≥11 months	20 (51.3%)	15 (57.7%)	5 (38.5%)	

^*^ *p*-value < .05 indicating statistically significant differences. ^**^ Staging according to the American Joint Committee on Cancer (AJCC) 8th edition.

^†^Median of 1 site of metastases (min 1; max 4).

^‡^Median of 3 treatment lines before performing LB.

^§^Median of 11 months of response to first-line systemic treatment to mCRC.

Abbreviations: CEA: carcinoembryonic antigen; ECOG PS: Eastern Cooperative Oncology Group Performance Status; LB: liquid biopsy; mCRC: metastatic colorectal cancer; RAS-mut: RAS mutated; RAS-wt: RAS wild type; WHO: World Health Organization.

The median age of the patients was 62 years (range 41 to 86) and 25 were men (64.0%). All participants had histologically confirmed adenocarcinoma, most commonly low-grade [26 (66.7%)], 13 (33.3%) of them with primary tumor located on left colon, 18 (46.2%) on right colon, and 8 (20.5%) in rectum. Twenty-three patients (59.0%) were diagnosed with stage IV disease. A median of one site of metastases (range 1 to 4) was detected at the time of diagnosis of mCRC, mainly hepatic (*n* = 25, 64.1%) and pulmonary (*n* = 12, 30.8%). One tumor had microsatellite instability and no *BRAF* mutation or *HER2* mutations or amplifications were detected.

Compared to the persistent *RAS-mut* group, patients in the *NeoRAS-wt* group were more likely to be diagnosed with early-stage disease (69.3% vs. 25.9% stage II-III CRC) and had more rectal adenocarcinomas (38.5% vs. 11.5%), higher number of metastatic sites (61.5% vs. 30.8% ≥2 metastatic sites), and lower CEA at the time of detection of metastatic disease (46.2% vs. 34.5% CEA < 3.5 ng/mL).

Curative-intent strategies were initially performed in 29 patients (74.4%), all including perioperative systemic therapy. All patients with early-stage disease (stages II or III) underwent surgery of primary tumor. For those presenting as mCRC, primary tumor surgery was performed in 22 patients (95.7%) [8 (36.4%) in context of intestinal occlusion] and complemented with metastasectomy in 12 (30.8%), the majority in the *RAS*-mut group (10 patients; 38.5% vs. 2 patients; 15.4%).

LB was performed after a median of 3 lines (range 0 to 7) of systemic treatment, earlier in patients with Neo*RAS-*wt (38.5% vs. 29.7% with <3 lines before LB). All patients had been previously treated with ChT and anti-VEGF Mabs.

At the time of LB collection, most patients had an ECOG PS 0-1 (*n* = 30, 76.9%), elevated CEA levels [CEA > 3.5 ng/mL in 32 patients (82.1%)] and <3 metastatic sites (*n* = 27, 69.2%), mainly in liver (*n* = 29, 74.4%) and lung (*n* = 25, 64.1%). Patients in Neo*RAS*-wt group tend to have better ECOG PS [ECOG PS 0-1 in 11 (84.6%) vs. 19 (73.1%)], but higher levels of CEA [CEA > 3.5 ng/mL in 9 (69.2%) vs. 23 patients (59.0%)] and higher number of metastatic sites (>2 sites in 5 (38.5%) vs. 7 (26.9%)], mostly in lung [10 (76.9%) vs. 15 (57.7%)], liver [9 (69.2%) vs. 20 (76.9%)] and peritoneum [4 (30.8%) vs. 8 (20.5%)] (Table 2).

**Table 2.** Demographic and clinical characteristics of the population at the time of LB collection.

Variable	Overall population (*n* = 39)	*RAS*-mut (*n* = 26)	Neo*RAS*-wt (*n* = 13)	*P*-value
ECOG PS at time of LB (*N*, %)				.689
0-1	30 (76.9%)	19 (73.1%)	11 (84.6%)	
>1	9 (23.1%)	7 (26.9%)	2 (15.4%)	
Number of metastatic sites^*^ (*N*, %)				.486
1-2 sites	27 (69.2%)	19 (73.1%)	8 (61.5%)	
>2 sites	12 (30.8%)	7 (26.9%)	5 (38.5%)	
Metastatic sites (*N*, %)				
Liver	29 (74.4%)	20 (76.9%)	9 (69.2%)	.704
Lung	25 (64.1%)	15 (57.7%)	10 (76.9%)	.304
Peritoneum	8 (20.5%)	4 (15.4%)	4 (30.8%)	.402
Other	16 (41.0%)	11 (42.3%)	5 (38.5%)	1.000
Level of CEA at time of LB (*N*, %)				.143
<3.5ng/mL	15 (38.5%)	9 (34.6%)	6 (46.2%)	
≥3.5 ng/mL	32 (82.1%)	23 (59.0%)	9 (69.2%)	
Missing	1 (0.02%)	1 (0.04%)	0 (00.0%)	

^*^Median of 2 sites of metastases (min 1; max 5).

Abbreviations: CEA: carcinoembryonic antigen; ECOG PS: Eastern Cooperative Oncology Group Performance Status; LB: liquid biopsy; mCRC: metastatic colorectal cancer; *RAS*-mut: *RAS* mutated; *RAS*-wt*: RAS* wild type.

### NeoRAS-wt Occurrence

Neo*RAS*-wt occurred in 13 patients (33.3%) and no *BRAF* mutations were detected in LB. Further treatment with anti-EGFR Mabs was administered to 9 (69.2%) patients (1 patient received anti-EGFR Mab in monotherapy and 8 patients received a combination of ChT and anti-EGFR Mab). The disease control rate was 44.4% (3 partial responses and 1 stable disease lasting 4.5 months), after a median of 6 cycles of treatment (range 4 to 20). The median duration of response was 3 months (range 2 to 52), and the median PFS on anti-EGFR mABs was 3 months (range 2 to 52). The evaluation of response to treatment was based on standard imaging, using RECIST criteria, without the requirement to repeat scanning in 3 to 4 weeks to confirm the response. Of the 9 patients treated, 1 patient with oligoprogression was then submitted to Stereotactic Body Radiation Therapy (SBRT) and 6 patients received further lines of systemic treatment [median of one line (range 1 to 3); 4 patients were rechallenged with duplet ChT plus bevacizumab, and 2 treated with TAS-102]. In later lines, regorafenib was administered to 3 of these 6 patients.

The association between clinical and pathological characteristics and *NeoRAS-wt* occurrence was studied with a binomial logistic regression model (see details in Statistical Analysis on Methods section). Two or more sites of metastases were the only variable positively associated with loss of *RAS-mut* (OR 6.08; 95% CI, 1.10-33.61; *P* = .038) (Table 3). However, this model is limited by the reduced number of patients in the study.

**Table 3.** Logistic regression model to Neo*RAS*-wt occurrence.

Variable	OR (95% CI)	*P*-value*
Two or more site of metastases	6.08 (1.10-33.61)	**.038**
Primary tumor of colon (right or left)	0.16 (0.23-1.13)	.067
Liver metastases	0.24 (0.47 -1.31)	.100

* p-value < .05 indicating statistically significant differences.Abbreviations: OR: Odds ratio; CI: confidence interval; wt: wild type.

### Survival

**Figure 2.**
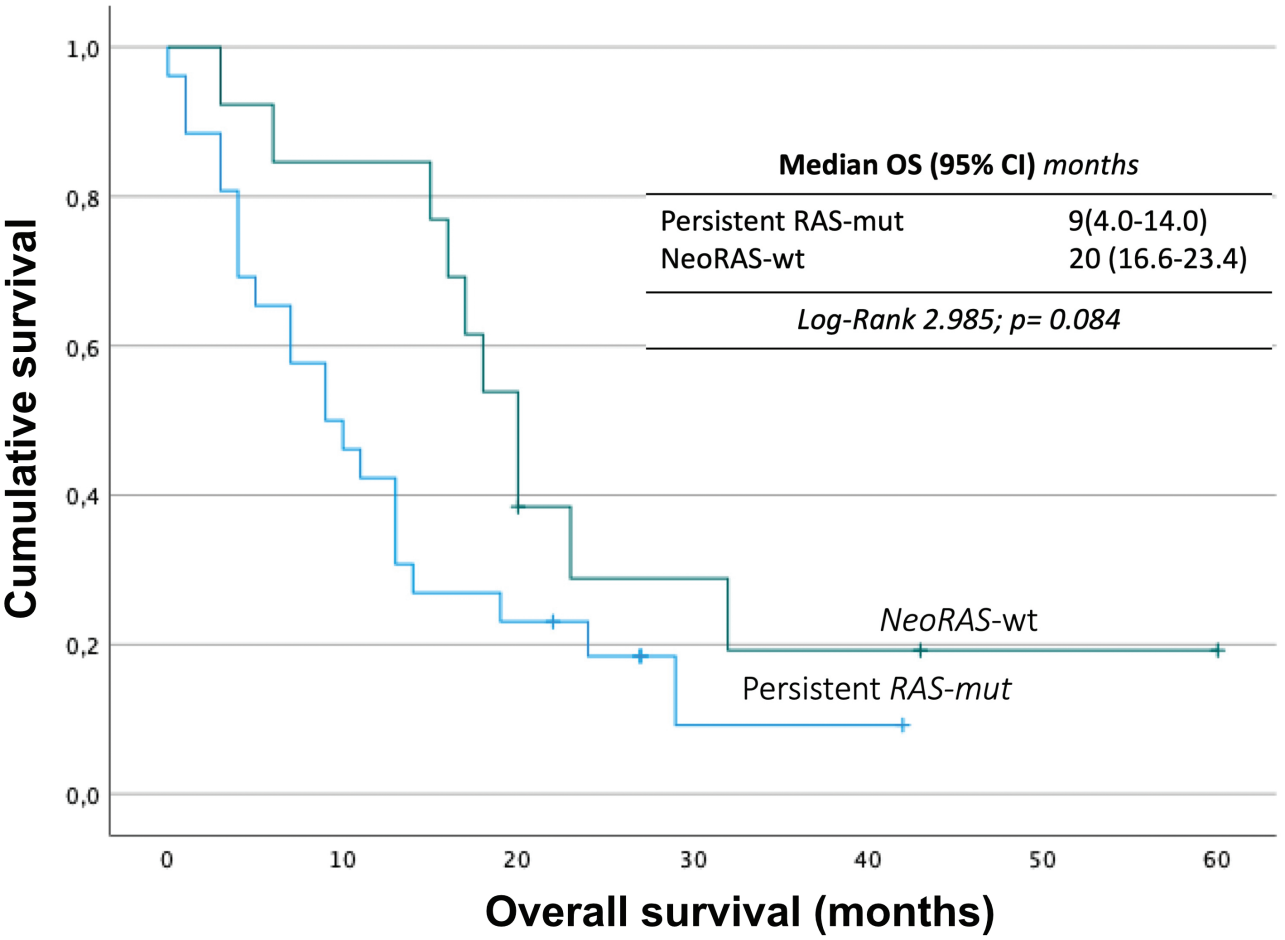
Survival analysis of persistent *RAS*-mut versus patients with Neo*RAS*-wt mCRC. Abbreviations: CI: confidence interval; OS: overall survival; *RAS-mut*: *RAS* mutated; RAS*-wt*: *RAS* wild-type.

At the time of database lock (June 6, 2023), the median follow-up for the patients who remained alive was 42 months (range 0 to 60). The median OS, from the date of LB testing, was 20 months (95% CI, 16.6-23.4) in the *NeoRAS-wt* group and 9 months (95% CI 4.0-14.0) in the persistent RAS-mut group (log-rank 2.985; *P* = .08) (Fig. 2), with a 12-month OS of 84.6% (95% CI, 74.6-94.6) and 57.7% (95% CI, 48.0-67.4), respectively.

Multivariate Cox regression analysis identified *NeoRAS-wt* as a predictor of survival [HR = 0.29 (95% CI, 0.12-0.71), *P* = .007]. Primary tumor of rectum [HR = 3.18 (95% CI, 1.13-8.92), *P* = .028] and peritoneal metastasis [HR = 2.95 (95% CI, 1.23-7.10), *P* = .160] were predictors of lower survival (Table 4). Other independent variables were tested (see details in Statistical Analysis on Methods section) but were removed from the final model due to lack of statistical significance.

**Table 4.** Multivariate Cox regression for overall survival.

Variable	HR (95% CI)	*P*-value*
*NeoRAS-wt*	0.29 (0.12-0.71)	.007
Peritoneal metastases	2.95 (1.29-7.10)	.016
Primary tumour of rectum	3.18 (1.13-8.92)	.02

*p-value < .05 indicating statistically significant differences.Abbreviations: HR: hazard ratio; CI: confidence interval; wt: wild type.

However, if OS is calculated from the date of diagnosis of metastatic disease, there is no difference between subgroups: 50 months for persistent *RAS*-mut versus 49 months for Neo*RAS*-wt (log-rank 0.097, *P* = .75).

## Discussion

In this study, we evaluated the prognostic value of *NeoRAS-wt* occurrence during the treatment course of patients with mCRC, while exploring patient and or tumor characteristics that could be correlated with *NeoRAS-wt*. In the analyzed cohort, *NeoRAS-wt* was observed in 13 patients (33.3%), 9 of which were further treated with anti-EGFR Mabs, usually associated with ChT, with a DCR of 44,4%. The only variable associated with Neo*RAS-*wt occurrence was higher tumor burden as accessed by the number of metastatic sites. However, this multivariate logistic regression analysis is limited by the low number of patients in the trial. The median OS in this population was 20 months and the 12-month OS was 84.6%. There was an 11-month difference in median OS between *NeoRAS*-wt and persistent *RAS-mut* subgroups, but this difference was not statistically different. However, in multivariate analysis, *NeoRAS*-wt occurrence was associated with a 71% risk reduction of death (*P* = .007).

Our results are in agreement with recent genomic data that established the pivotal role of *RAS-mut* in selecting the most active/effective treatment options for mCRC.^5,6,12,16,21,22^ By resorting to ctDNA analysis by LB, these studies evidenced the loss of *RAS* mutant clones under ChT pressure, by changing to Neo*RAS*-wt,^6^ and reported evidence of negative selection of *RAS*-mut during the clonal evolution of mCRC.^5,7,12,21^ Regarding the treatment of Neo*RAS*-wt mCRC, our results also agreed with those reported in recent studies. In fact, 4 of 9 patients with *NeoRAS-wt* LB who received anti-EGFR Mabs achieved clinical benefit, as described before by Gazzaniga et al, in a case report published in 2018.^23^ Similar results have been reported in small series of patients with *NeoRAS*-wt treated with anti-EGFR Mabs, with prolongation of PFS,^12^ OS, and clinical response.^16,21^

In this study, we observed a lower rate of occurrence of Neo*RAS*-wt (33.3%) than those reported previously.^5,12^ This trend may be related to different timings of LB collection, variable disease burden, heterogeneous previous lines of treatment, and analytical sensitivity of Idylla. Regardless, the treatment of patients with Neo*RAS*-wt with anti-EGFR Mabs resulted in promising results, with identification of Neo*RAS*-wt (HR = 0.29, *P* = .006) as a positive predictor of survival, with a risk reduction of death of 71.0%. Presence of peritoneal metastases and primary tumor of rectum were associated with worse prognosis and shorter survival (*P* < .050). Our findings support that dynamic monitoring of the clonal evolution of mCRC by LB may open new treatment opportunities.^5,24^ In fact, after failure of standard-of-care regimens and retesting for *RAS* mutational status, the use of anti-EGFR Mabs may provide additional clinical benefit, as shown, herein and in recent studies. However, other patient and tumor characteristics may have contributed to the better survival observed in these patients.^25^ Even though the optimal timing for retesting has not yet been defined, current recommendations include the evaluation of *RAS* mutational status not only before starting first-line treatment but also after the failure of early treatment lines.

Although our cohort represents one of the largest case series reported so far, sample size, the retrospective nature of the study, single ctDNA time point evaluation with subsequent limited plasma genomic sequencing of variant allele frequency of *RAS* gene, and evaluation of molecular changes only in *RAS* and *BRAF* genes are relevant limitations of the study. Also, the analytical sensitivity of Idylla can result in false negative results in patients with variant allele frequency below 1%, in which the absence of detectable *RAS* mutations in plasma cannot certainly exclude the presence of a *RAS* mutation below the assay limit of detection. The use of NGS instead of Idylla would have improved the sensitivity of the ctDNA evaluation. In addition, performing a tissue biopsy and molecular analyses in patients with Neo*RAS*-wt to confirm the result of LB would also be clarifying. The results of ongoing prospective studies will shed further light on this topic.^6,26-28^

In conclusion, our study showed that monitoring *RAS* clonal alterations by LB, in patients with mCRC, may allow additional effective treatments for patients with Neo*RAS*-wt with advanced disease, with clinical and survival benefits.

## Data Availability

The data underlying this article will be shared on reasonable request to the corresponding author.
